# Associations of quantitative whole-body PSMA-PET metrics with PSA progression status under long-term androgen deprivation therapy in prostate cancer patients: a retrospective single-center study

**DOI:** 10.1186/s41824-023-00178-1

**Published:** 2023-10-02

**Authors:** Vishnu Murthy, Emmanuel Appiah-Kubi, Kathleen Nguyen, Pan Thin, Masatoshi Hotta, John Shen, Alexandra Drakaki, Matthew Rettig, Andrei Gafita, Jeremie Calais, Ida Sonni

**Affiliations:** 1 Ahmanson Translational Theranostics Division, Department of Molecular and Medical Pharmacology, David Geffen School of Medicine at UCLA, 10833 Le Conte Avenue, Los Angeles, CA 90095 USA; 2 Division of Hematology/Oncology, Department of Medicine, David Geffen School of Medicine at UCLA, Los Angeles, CA USA; 3 Department of Radiological Sciences, David Geffen School of Medicine at UCLA, Los Angeles, CA USA; 4 Department of Experimental and Clinical Medicine, Magna Graecia University, Catanzaro, Italy

**Keywords:** PSMA-PET, ADT, ARSi, RECIP

## Abstract

**Purpose:**

To evaluate whether quantitative whole-body (WB) PSMA-PET metrics under long-term androgen deprivation therapy (ADT) and/or androgen receptor signaling inhibitors (ARSi) are associated with PSA progression.

**Methods:**

Patients who underwent at least 2 ^68^Ga-PSMA-11 PET/CT scans between October 2016 and April 2021 (*n* = 372) and started a new line of ADT ± ARSi between PET1 and PET2 were retrospectively screened for inclusion. We investigated the association between PCWG3-defined PSA progression status at PET2 and the following PSMA-PET parameters: appearance of new lesions on PET2, ≥ 20% increase in WB-PSMA tumor volume (WB-PSMA-VOL), progression of disease (PD) by RECIP 1.0, and ≥ 30% increase in WB-PSMA-SUV_mean_ from PET1 to PET2. Spearman’s rank correlation coefficients and Fisher’s exact test were used to evaluate the associations.

**Results:**

Thirty-five patients were included: 12/35 (34%) were treated with ADT only and 23/35 (66%) with ARSi ± ADT. The median time between PET1 and PET2 was 539 days. Changes (%) in median PSA levels, WB-PSMA-SUV_mean_, and WB-PSMA-VOL from PET1 to PET2 were -86%, -23%, and -86%, respectively. WB-PSMA-VOL ≥ 20%, new lesions, RECIP-PD, and WB-PSMA-SUV_mean_ ≥ 30% were observed in 5/35 (14%), 9/35 (26%), 5/35 (14%), and 4/35 (11%) of the whole cohort, in 3/9 (33%), 7/9 (78%), 3/9 (33%), and 2/9 (22%) of patients with PSA progression at PET2, and in 2/26 (8%), 2/26 (8%), 2/26 (8%), and 2/26 (8%) of patients without PSA progression at PET2 (*p* = 0.058, *p* < 0.001, *p* = 0.058, *p* = 0.238, respectively). Changes in PSA were correlated to percent changes in WB-PSMA-VOL and WB-PSMA-SUV_mean_ (Spearman ρ: 0.765 and 0.633, respectively; *p* < 0.001).

**Conclusion:**

Changes in PSA correlated with changes observed on PSMA-PET, although discordance between PSA and PSMA-PET changes was observed. Further research is necessary to evaluate if PSMA-PET parameters can predict progression-free survival and overall survival and serve as novel endpoints in clinical trials.

**Supplementary Information:**

The online version contains supplementary material available at 10.1186/s41824-023-00178-1.

## Introduction

Prostate cancer (PCa) is the most frequently diagnosed malignancy in men in developed countries and a leading cause of cancer death worldwide (Sung et al. 2021). Since the discovery of the androgen dependence of PCa cells, treatments aiming at suppressing testosterone levels have represented the main systemic therapy approach for patients with advanced regional or metastatic disease (Schaeffer et al. 2021). Patients who continue to experience a rise in serum PSA levels while on androgen deprivation therapy (ADT) are classified as castration-resistant (CRPC), and treatment with second-line androgen receptor signaling inhibitors (ARSi), such as abiraterone and enzalutamide, is considered. Several studies have demonstrated the efficacy of ARSi in treating metastatic CRPC (mCRPC) (Beer et al. 2014; Bono et al. 2011), and more recently, these treatments were also shown to be useful in patients with metastatic castration-sensitive PCa (mCSPC) (Tombal et al. 2014; Fizazi et al. 2019).

Prostate-specific membrane antigen (PSMA) is a transmembrane glycoprotein that is significantly upregulated in PCa cells (Silver et al. 1997). This characteristic makes PSMA a target for molecular imaging and radioligand therapy (RLT) of PCa and led to the development of several radiopharmaceuticals for nuclear theranostics applications (Lutje et al. 2017). Due to high diagnostic accuracy and detection rates, positron emission tomography (PET) targeting PSMA (PSMA-PET) is now a well-established imaging tool in the evaluation of both primary and recurrent PCa (Hofman et al. 2020; Fendler et al. 2019).

Further research is necessary to characterize the relationship between PSMA expression and ADT/ARSi initiation and understand how changes in PSMA-PET features correlate with clinical response criteria in PCa patients initiating treatment with ADT/ARSi. Currently available literature on the relationship between PSMA expression and ADT/ARSi initiation suggests that short-term ADT may increase PSMA uptake, while continuous, long-term ADT may reduce PSMA uptake (Vaz et al. 2020). However, this literature is highly heterogeneous in terms of cohort size, castration status, and type/duration of ADT.

Recently, response evaluation criteria in PSMA-PET/CT (RECIP) 1.0 were introduced (Gafita et al. 2022a). Patients were classified as having progressive disease (PD) if they experienced a ≥ 20% increase in whole-body (WB) PSMA tumor volume (WB-PSMA-VOL) and had new lesions on interim PSMA-PET done after two cycles of ^177^Lu-PSMA RLT. Progression on interim PSMA-PET by RECIP 1.0 was shown to be prognostic for overall survival (OS) in mCRPC patients undergoing treatment with ^177^Lu-PSMA RLT (Gafita et al. 2022a). While previous response criteria have primarily relied on qualitative lesion-based assessments (appearance of new lesions), RECIP 1.0 employs both WB quantitative PSMA-PET parameters and lesion-based analyses. However, RECIP 1.0 has not previously been studied outside of mCRPC patients undergoing treatment with RLT. The aim of this retrospective, single-center study was to evaluate whether changes in WB-PSMA-PET metrics under long-term ADT/ARSi are associated with PSA progression.

## Methods

### Patients

Patients who underwent at least 2 ^68^Ga-PSMA-11 PET/CT scans between October 2016 and April 2021, with at least one scan done at UCLA as part of a prospective clinical trial (NCT03792841, NCT03515577, NCT04050215, NCT02940262, NCT04348682, NCT04282824, NCT03368547, NCT03042312, NCT03582774, and NCT03511664) or clinically after FDA approval of ^68^Ga-PSMA-11, were retrospectively screened for this study. Due to the retrospective design, this study was approved by the institutional review board with a waiver of informed consent (IRB#20-000954). Patients who started a new line of ADT and/or ARSi between PET1 and PET2 were included. Both CSPC and CRPC patients could be included, as well as patients with non-metastatic and metastatic disease. Patients starting any other PCa-related treatment between the two PSMA-PET scans, patients who suspended ADT/ARSi within 30 days of initiation, and patients without clinical follow-up data were excluded. Median time under ADT/ARSi was calculated as the time from ADT/ARSi initiation to treatment cessation or PET2, if treatment was ongoing at the time of PET2. Clinical information was extracted from electronic medical records by two of the investigators.

### PSMA-PET acquisition and image analysis

^68^Ga-PSMA-11 PET/CT image acquisition has been previously described (Calais et al. 2018). The image interpretation and analysis were performed by a board-certified nuclear medicine physician, blinded to PSA progression status and unblinded to the clinical PSMA-PET report. The WB-PSMA-SUV_mean_ and WB-PSMA-VOL were obtained using qPSMA software as previously described (Gafita et al. 2019). The presence of new lesions on PET2 and PROMISE molecular imaging TNM (miTNM) staging were evaluated using OsiriX Lite (version 13.0.1) (Rosset et al. 2004). The PROMISE miTNM system is a standardized reporting framework for PSMA-PET that is analogous to TNM staging based on clinicopathologic variables (Eiber et al. 2018).

### WB-PSMA-PET metrics and clinical outcomes

We investigated the associations between PSA progression status defined using Prostate Cancer Clinical Trials Working Group 3 (PCWG3) criteria (Scher et al. 2016) at time of PET2 and the following imaging parameters: baseline WB-PSMA-VOL, baseline WB-PSMA-SUV_mean,_ changes in miTNM staging from PET1 to PET2, appearance of new lesions on PET2, ≥ 20% increase in WB-PSMA-VOL from PET1 to PET2, RECIP-PD (≥ 20% increase in WB-PSMA-VOL and appearance of new lesions on PET2), and ≥ 30% increase in WB-PSMA-SUV_mean_ from PET1 to PET2. We used a 20% cutoff for changes in WB-PSMA-VOL and a 30% cutoff for changes in WB-PSMA-SUV_mean_ to be consistent with RECIP 1.0 and PERCIST, respectively (Gafita et al. 2022a; Wahl et al. 2009).

### Statistical analysis

Fisher’s exact test was used to assess the significance of the associations between PSMA-PET parameters and PSA progression status at PET2. Spearman’s rank correlation coefficients were generated to evaluate the association of percent changes in WB-PSMA-VOL and WB-PSMA-SUV_mean_ with percent changes in PSA between PET1 and PET2. Group descriptive statistics are expressed in median and interquartile range (IQR) unless stated otherwise. Statistical analysis was done using Jamovi (The Jamovi Project [computer program] 2021).

## Results

### Patients

**Fig. 1:**
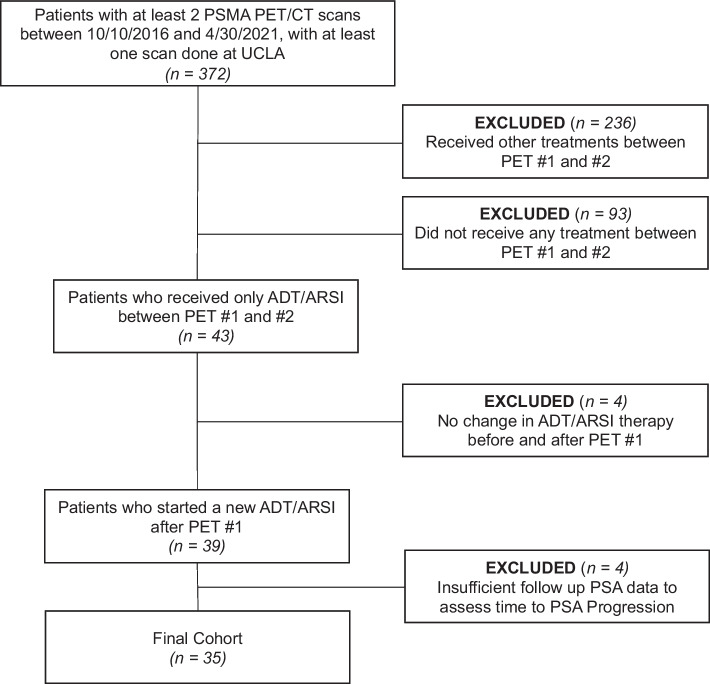
Patient selection flowchart. A total of thirty-five patients were included in the final cohort

Among 372 patients who underwent 2 PSMA-PET scans between 10/10/2016 and 4/30/2021, 35 patients were included in the analysis. The patient selection flowchart is shown in Fig. 1. Table 1 outlines patient demographics, treatment characteristics, and reason for PET2 referral. Thirty-three patients were still alive at the time of our analysis. 12/35 patients (34%) were treated with ADT alone and 23/35 (66%) were treated with ARSi ± ADT. 29/35 patients (83%) were castration-sensitive at PET1, while 6/35 (17%) were castration-resistant. 5/35 (14%) of patients had an miTNM stage of T0N0M0 at baseline, 2/35 (6%) had prostate/prostate fossa only (TrN0M0), 12/35 (34%) had nodal disease only (miTxN1/M1a), 3/35 (9%) had bone disease only (TxNxMlb), 8/35 (23%) had both nodal and bone disease (TxN1/Mla Mlb), and 5/35 (14%) had visceral metastatic disease (TxNxM1c). Patient-based reporting of Gleason Score, castration status, classification of PD vs. non-PD based on RECIP 1.0, and percent changes in WB-PSMA-VOL and WB-PSMA-SUV_mean_ is also provided in Additional file 1.

**Table 1:** Patient demographics and treatment characteristics

Number of patients	35
Median age, years (IQR)	70 (66.5–74)
Gleason score (from prostatectomy when available)	
3 + 3 = 6	2
3 + 4 = 7	8
4 + 3 = 7	2
3 + 5 = 8	1
4 + 4 = 8	5
4 + 5 = 9	8
5 + 4 = 9	5
5 + 5 = 10	4
Initial treatment, *n* (%)	
Surgery	17 (48.6)
Radiation therapy	8 (22.9)
Hormonal treatment	9 (25.7)
Other	1 (2.9)
*Type of treatment added after PET1, n (%)*	
ADT	
Lupron	4 (11.4)
Casodex	4 (11.4)
Zoladex	1 (2.9)
Lupron + Casodex	3 (8.6)
ARSi ± ADT	
Abiraterone	5 (14.3)
Enzalutamide	2 (5.7)
Apalutamide	3 (8.6)
Lupron + Casodex + Darolutamide	1 (2.9)
Lupron + Abiraterone	10 (28.6)
Lupron + Apalutamide	1 (2.9)
Lupron + Enzalutamide	1 (2.9)
Castration status at PET1, *n* (%)	
Castration-sensitive	29 (82.9)
Castration-resistant	6 (17.1)
Median Days Under ADT/ARSi (IQR)	324 (221–550)
Patients on ADT/ARSi ≤ 90 days, *n* (%)	1 (2.9)
Patients on ADT/ARSi between 90 and 180 days, *n* (%)	6 (17.1)
Patients on ADT/ARSi between 180 and 365 days, *n* (%)	15 (42.9)
Patients on ADT/ARSi ≥ 365 days, *n* (%)	13 (37.1)
Reason for PET2 referral	
Localization of biochemical recurrence, *n* (%)	14 (40)
Therapy response assessment, *n* (%)	3 (8.6)
Subsequent treatment strategy, *n* (%)	4 (11.4)
Restaging after multiple therapies, *n* (%)	12 (34.3)
Other, *n* (%)	2 (5.7)
Median days between PET1 and PET2 (IQR)	539 (355.5–802)
Median days between PET1 and ADT/ARSi initiation (IQR)	26 (6.5–79.5)
Median days between ADT/ARSi Initiation and PET2 (IQR)	380 (248–617.5)
Median days to PSA progression from ADT/ARSi initiation (IQR)	1022 (708.5–1348.5)

The median time between PET1 and ADT/ARSi initiation was 26 days (IQR: 6.5–79.5). The median time between ADT/ARSi initiation and PET2 was 380 days (IQR: 248–617.5). The median time under ADT/ARSi was 324 days (IQR: 221–550), and the median time between PET1 and PET2 was 539 days (IQR: 355.5–802).

### PSMA-PET metrics and PSA outcomes

Table 2 summarizes changes in PSA, miTNM staging, WB-PSMA-SUV_mean_, and WB-PSMA-VOL between PET1 and PET2. The percent changes between PET1 and PET2 in median serum PSA levels, WB-PSMA-SUV_mean_, and WB-PSMA-VOL were − 86%, − 23%, and − 86%, respectively.

**Table 2:** Changes in PSA, miTNM stage, WB-PSMA-SUV_mean_, and WB-PSMA-VOL between PET1 and PET2

	PET1	PET2	% Change
Median PSA, ng/mL (range)	4.4 (0.02–336.9)	0.61 (0–95.1)	− 86.1
miTNM, *n* (%)			N/A
T0N0M0	5 (14.3)	10 (28.6)	
TrN0M0	2 (5.7)	2 (5.7)	
N1 and / or M1a (LN disease only)	12 (34.3)	9 (25.7)	
M1b (bone disease only)	3 (8.6)	4 (11.4)	
N1 and/or M1a and M1b (LN + Bone disease)	8 (22.9)	7 (20)	
M1c	5 (14.3)	3 (8.6)	
Median WB-PSMA-SUV_mean_	4.993 (0–13.023)	3.827 (0–11.959)	− 23.4
Median WB-PSMA-VOL (range)	42.404 (0–1501.644)	5.879 (0–2371.702)	− 86.1

Percent changes in PSA were correlated to percent changes in WB-PSMA-VOL and WB-PSMA-SUV_mean_ (Spearman ρ: 0.765 and 0.633, respectively; *p* < 0.001). Percent changes in PSA were also correlated to percent changes in WB-PSMA-VOL and WB-PSMA-SUV_mean_ when analyzing castration-sensitive patients (Spearman *ρ* 0.819 and 0.712, respectively; *p* < 0.001), although a similar relationship was not observed when analyzing castration-resistant patients (Spearman *ρ* 0.469 and 0.462, respectively; *p* = 0.348 and *p* = 0.356, respectively).

**Fig. 2:**
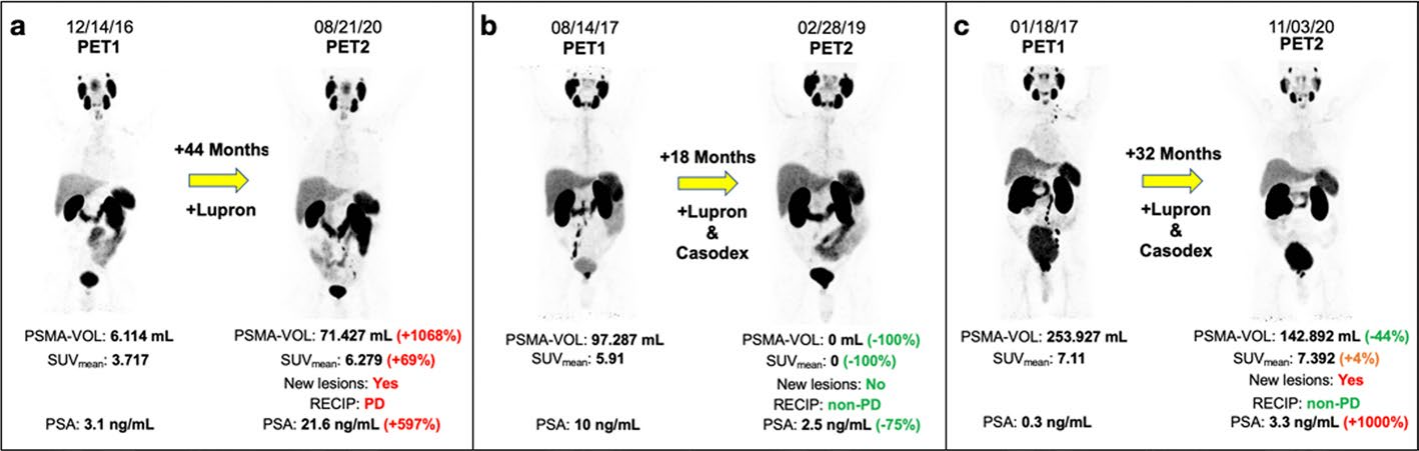
**a** PSA and PSMA-PET metrics concordance: 75-year-old male with Gleason Score 3 + 4 = 7 castration-sensitive PCa treated with Lupron between PET1 and PET2. Patient had WB-PSMA-VOL ≥ 20%, WB-PSMA-SUV_mean_ ≥ 30%, was classified as RECIP-PD, had new lesions on PET2, and experienced PSA progression at the time of PET2. **b** PSA and PSMA-PET metrics concordance: 66-year-old male with Gleason Score 3 + 4 = 7 castration-sensitive PCa treated with Lupron and Casodex between PET1 and PET2. Patient had WB-PSMA-VOL < 20%, WB-PSMA-SUV_mean_ < 30%, was classified as RECIP-non-PD, had no new lesions on PET2, and did not experience PSA progression at the time of PET2. **c** PSA and PSMA-PET metrics discordance: 70-year-old male with Gleason Score 5 + 5 = 10 castration-sensitive PCa treated with Lupron and Casodex between PET1 and PET2. Patient had WB-PSMA-VOL < 20%, WB-PSMA-SUV_mean_ < 30%, was classified as RECIP-non-PD, had new lesions on PET2, but did experience PSA progression at the time of PET2

Overall, 9/35 (26%) patients experienced PSA progression at time of PET2 and 26/35 (74%) patients did not. Table 3 summarizes the associations of PSMA-PET characteristics with PSA progression status at PET2. Figure 2 shows sample cases from our cohort.

**Table 3:** PSMA-PET metrics and PSA outcomes

	Total	PSA progression at PET2	No PSA progression at PET2	*P* value
Total, *n* (%)	35 (100)	9 (25.7)	26 (74.3)	N/A
Castration status, *n* (%)				0.304
Castration-sensitive, *n* (%)	29 (82.9)	9 (100)	20 (76.9)	
Castration-resistant, *n* (%)	6 (17.1)	0 (0)	6 (23.1)	
miTNM Stage (PET1–PET2)				0.007
Upstaged, *n* (%)	8 (22.9)	5 (55.6)*	3 (11.5)	
Downstaged/no change, *n* (%)	27 (77.1)	4 (44.4)	23 (88.5)	
Baseline WB-PSMA-VOL				0.009
Above median, *n* (%)	18 (51.4)	8 (88.9)*	10 (38.5)	
Below median, *n* (%)	17 (48.6)	1 (11.1)	16 (61.5)	
Changes in WB-PSMA-VOL				0.058
≥ 20%, *n* (%)	5 (14.3)	3 (33.3)	2 (7.7)	
< 20%, *n* (%)	30 (85.7)	6 (66.7)	24 (92.3)	
New Lesions on PET2				< .001
Yes, *n* (%)	9 (25.7)	7 (77.8)*	2 (7.7)	
No, *n* (%)	26 (74.3)	2 (22.2)	24 (92.3)	
RECIP 1.0				0.058
PD, *n* (%)	5 (14.3)	3 (33.3)	2 (7.7)	
Non-PD, *n* (%)	30 (85.7)	6 (66.7)	24 (92.3)	
Baseline WB-PSMA-SUV_mean_				0.009
Above median, *n* (%)	18 (51.4)	8 (88.9)*	10 (38.5)	
Below median, *n* (%)	17 (48.6)	1 (11.1)	16 (61.5)	
Changes in WB-PSMA-SUV_mean_				0.238
≥ 30%, *n* (%)	4 (11.4)	2 (22.2)	2 (7.7)	
< 30%, *n* (%)	31 (88.6)	7 (77.8)	24 (92.3)	

*Significant by Fisher’s exact test

*Baseline PSMA (PET1) and PSA progression:* 8/9 (89%) patients with PSA progression at PET2 had a baseline WB-PSMA-VOL and WB-PSMA-SUV_mean_ above the median compared with 10/26 (38%) patients without PSA progression at PET2 (*p* = 0.009).

**Fig. 3:**
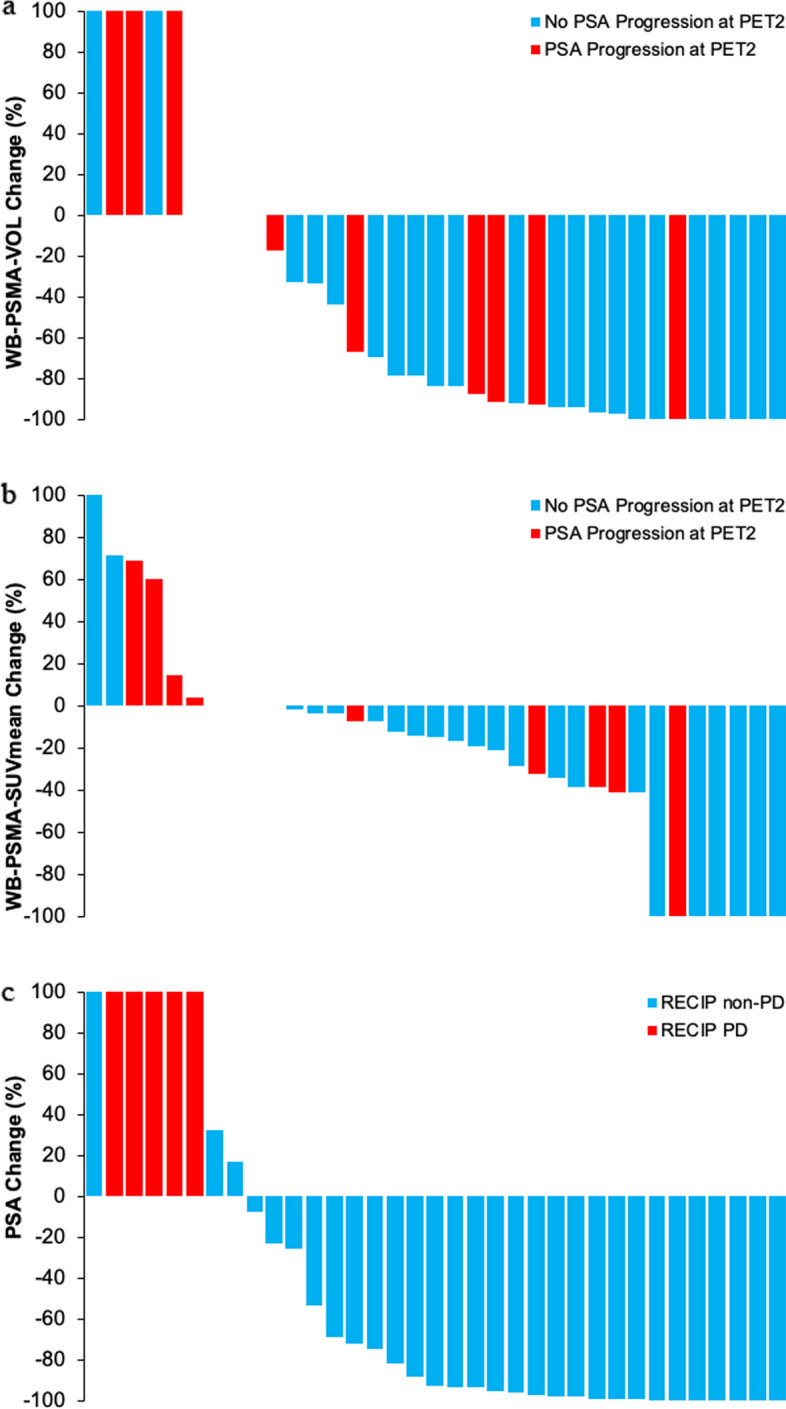
Waterfall plots depicting the relationship between percent changes in WB-PSMA-VOL and PSA progression status at PET2 (**a**), percent changes in WB-PSMA-SUV_mean_ and PSA progression status at PET2 (**b**), and percent changes in PSA and RECIP 1.0 progression status (**c**)

*Follow-up PSMA (PET2) and PSA progression:* upstaging by miTNM criteria occurred in 8/35 (23%) of the whole cohort, 5/9 (56%) of the patients with PSA progression at PET2, and 3/26 (12%) of patients without PSA progression at PET2 (*p* = 0.007). Associations between castration status, WB-PSMA-VOL, new lesions, RECIP 1.0, WB-PSMA-SUV_mean_, and PSA progression status at PET2 are summarized in Table 3. Waterfall plots depicting the relationship between percent changes in WB-PSMA-VOL and PSA progression status at PET2, percent changes in WB-PSMA-SUV_mean_ and PSA progression status at PET2, and percent changes in PSA and RECIP 1.0 progression status are shown in Fig. 3.

## Discussion

This single-center retrospective study evaluated changes in quantitative WB-PSMA-PET parameters in patients treated with long-term ADT/ARSi. Overall, between PET1 (prior to treatment initiation) and PET2 (after treatment), we observed a decrease in the volume of disease quantified on PSMA-PET (WB-PSMA-VOL) and mean whole-body PSMA uptake (WB-PSMA-SUV_mean_). Our findings are consistent with those of previously published retrospective studies showing that continuous long-term ADT significantly reduces the PSMA expression of castration-sensitive PCa (Afshar-Oromieh et al. 2018; Hoberuck et al. 2020). Other studies also found significant decreases at 3 months in the PSMA-PET SUV_max_ of primary tumor and lymph nodes in patients treated with short-term ADT (Onal et al. 2020; Tseng et al. 2022).

A PSMA flare phenomenon, describing an increase in the quantitative measure of PSMA expression (SUV_mean_ or SUV_max_) during the first week or month following initiation of ADT/ARSI, has previously been described (Hope et al. 2017; Aggarwal et al. 2018). This phenomenon was not investigated in our analysis due to the longer time duration of the ADT/ARSi treatment in our cohort, with a median time under ADT/ARSi of 324 days (IQR: 221–550) and a median time interval between PET1 and PET2 of 539 days (IQR: 355.5–802). Our study rather assessed the impact of long-term ADT/ARSi treatment on quantitative PSMA-PET metrics and evaluated whether they are associated with PSA progression.

In this analysis, percent changes in PSA were correlated with percent changes in WB-PSMA-VOL and WB-PSMA-SUV_mean_, suggesting that changes in quantitative PSMA-PET metrics may be predictive of clinical response. These findings are consistent with another retrospective analysis of 44 mCRPC patients, which found a concordance between changes in WB-PSMA-PET derived parameters and changes in PSA (Oruc et al. 2021). Our analysis also shows that patients who were upstaged by miTNM staging on PET2 were more likely to experience PSA progression on PET2, suggesting that miTNM staging using PROMISE criteria is associated with conventional response criteria. Further research is necessary to characterize the role of miTNM staging in the prognosis and management of PCa. Furthermore, patients with a higher baseline WB-PSMA-VOL and WB-PSMA-SUV_mean_ in our cohort were more likely to experience PSA progression at PET2. Zukotynski et al. report that in a cohort of 16 men with mCRPC, patients with a low number of lesions on their baseline ^18^F-DCFPyL PET/CT had longer OS, although the difference was not statistically significant (*p* = 0.35) (Zukotynski et al. 2021). The results from our analysis suggest that in patients undergoing treatment with ADT/ARSi, baseline quantitative metrics on PSMA-PET may be associated with conventional response criteria, although this conclusion requires further validation in larger, prospective trials.

Patients who experienced PSA progression on PET2 were also more likely to be classified as RECIP-PD. These findings are consistent with Plouznikoff et al., who found that in patients treated with ARSi, PSMA response was associated with conventional response criteria (PSA and RECIST) (Plouznikoff et al. 2019). Furthermore, in a prospective, single-arm trial of mCRPC patients initiating abiraterone or enzalutamide who underwent follow-up ^18^F-DCFPyL PET/CT within 2–4 months of treatment initiation, the sum of percent and absolute changes in SUV_max_ for all positive lesions were associated with OS (Zukotynski et al. 2021). It should also be noted that while patients who experienced PSA progression at PET2 had a change in WB-PSMA-VOL ≥ 20% or were classified as RECIP-PD at higher rates than patients who did not experience PSA progression, this difference was not statistically significant (*p* = 0.058), possibly due to the small sample size in this cohort. It should also be noted that RECIP 1.0 was initially studied in mCRPC patients undergoing treatment with RLT, while our analysis studies RECIP 1.0 in patients undergoing treatment with ADT/ARSi. Further research is necessary in larger, prospective trials across multiple clinical settings to evaluate the association between progression on PSMA-PET by RECIP 1.0 and PSA progression status, metastasis-free survival, and overall survival.

It should also be noted that the appearance of new lesions on PET2 as a single lesion assessment does not fully capture disease heterogeneity. In our analysis, while 7/9 patients who experienced PSA progression at PET2 had new lesions on PET2, only 5/9 patients were upstaged based on miTNM staging and 3/9 patients were classified as PD based on changes in WB-PSMA-VOL or RECIP 1.0. This discrepancy illustrates the importance of using whole-body PET parameters to assess treatment response. In a comparative analysis of criteria for therapy response assessment in mCRPC, RECIP 1.0 identified fewer patients with PD, and patients classified as PD by RECIP 1.0 had a higher risk of death than non-PD patients compared to lesion-based criteria (Gafita et al. 2022b). This suggests that lesion-based criteria may overcall progression and that changes in quantitative, whole-body PSMA-PET parameters may better reflect changes in metastatic prostate cancer in patients undergoing systemic therapy (Gafita et al. 2022b). Segmentation methods are currently under development to ensure the fast, reproducible, and widespread use of WB-PET metrics in clinical practice (Gafita et al. 2019; Seifert et al. 2020).

While there was a general concordance between PSA progression status at PET2 and PSMA-PET findings, there were six patients who were classified as non-PD by RECIP 1.0 who experienced PSA progression at PET2, while there were two patients who were classified as PD by RECIP 1.0 who did not experience PSA progression at PET2. In the RECIP 1.0 study, among patients without a PSA response at 12 weeks (76/124, 61%), patients classified as having a partial response by RECIP 1.0 (10/76, 13%) had a longer OS compared to patients without a partial response (66/76, 87%): 22.7 versus 9.0 months (Gafita et al. 2022a). Similarly, among patients without PSA progression at 12 weeks (84/124, 68%), patients classified as PD by RECIP 1.0 (12/84, 14%) had a shorter OS compared to patients classified as non-PD (72/84, 86%): 7.7 vs 18.1 months (Gafita et al. 2022a). These results demonstrate the added value of PSMA-PET findings in patients who may not be differentiated by conventional biomarkers, such as PSA. Given the small size of our cohort, we were not able to directly compare PSA and PSMA-PET in a similar manner.

The main limitation of this retrospective study is the selection bias: patients were referred for PET2 for re-staging and/or recurrence based on PSA elevation. Future clinical trials should include PSMA-PET assessments systematically regardless of whether patients progress or not. Another main limitation is the absence of analysis of OS and PSA PFS. 94% of our cohort was still alive at the time of analysis and 26% experienced PSA progression prior to PET2. Further research in larger, prospective trials is necessary to correlate PSMA-PET parameters directly with PSA PFS and OS in patients undergoing treatment with ADT/ARSi. Other limitations include a heterogeneous population with both CSPC and CRPC patients, as well as patients treated with both ADT and/or ARSi. The small sample size did not allow us to stratify patients based on prior treatment or disease state. Finally, the use of Fisher’s exact test in a smaller cohort may also have lower power to disprove a null hypothesis.

PSMA-PET allows clinicians to identify the location and define the extent of disease burden with superior accuracy compared to conventional imaging. PSA increases may occur due to cancer cell death in the absence of clinically meaningful disease progression. Therefore, assessing radiographic progression using PET molecular imaging should be considered in the management of patients in addition to PSA to guide treatment decisions in a more personalized manner. Quantitative response assessment also holds clinical benefit when assessing response in osseous lesions. By assessing progression by PSMA-PET using WB quantitative measures, we can determine in a more precise manner whether osseous metastases have improved, while conventional imaging can only inform us of stable disease vs. disease progression.

## Conclusion

In this retrospective analysis, changes in PSA correlated with changes observed on PSMA-PET, although discordance between PSA and PSMA-PET changes was observed. Further research is necessary to evaluate if PSMA-PET parameters can predict PFS and OS and serve as novel endpoints in clinical trials.

### Supplementary Information

**Additional file 1. **Supplementary Materials.

## Data Availability

The data that support the findings of this study are available from the corresponding author, JC, upon reasonable request.

## References

[CR1] Afshar-Oromieh A, Debus N, Uhrig M (2018). Impact of long-term androgen deprivation therapy on PSMA ligand PET/CT in patients with castration-sensitive prostate cancer. Eur J Nucl Med Mol Imaging.

[CR2] Aggarwal R, Wei X, Kim W (2018). Heterogeneous flare in prostate-specific membrane antigen positron emission tomography tracer uptake with initiation of androgen pathway blockade in metastatic prostate cancer. Eur Urol Oncol.

[CR3] Beer TM, Armstrong AJ, Rathkopf DE (2014). Enzalutamide in metastatic prostate cancer before chemotherapy. N Engl J Med.

[CR4] Calais J, Kishan AU, Cao M (2018). Potential impact of (68)Ga-PSMA-11 PET/CT on the planning of definitive radiation therapy for prostate cancer. J Nucl Med.

[CR5] de Bono JS, Logothetis CJ, Molina A (2011). Abiraterone and increased survival in metastatic prostate cancer. N Engl J Med.

[CR6] Eiber M, Herrmann K, Calais J (2018). Prostate cancer molecular imaging standardized evaluation (PROMISE): proposed miTNM classification for the interpretation of PSMA-ligand PET/CT. J Nucl Med.

[CR7] Fendler WP, Calais J, Eiber M (2019). Assessment of 68Ga-PSMA-11 PET accuracy in localizing recurrent prostate cancer: a prospective single-arm clinical trial. JAMA Oncol.

[CR8] Fizazi K, Tran N, Fein L (2019). Abiraterone acetate plus prednisone in patients with newly diagnosed high-risk metastatic castration-sensitive prostate cancer (LATITUDE): final overall survival analysis of a randomised, double-blind, phase 3 trial. Lancet Oncol.

[CR9] Gafita A, Bieth M, Kronke M (2019). qPSMA: semiautomatic software for whole-body tumor burden assessment in prostate cancer using (68)Ga-PSMA11 PET/CT. J Nucl Med.

[CR10] Gafita A, Rauscher I, Weber M (2022). Novel framework for treatment response evaluation using PSMA-PET/CT in patients with metastatic castration-resistant prostate cancer (RECIP 1.0): an international multicenter study. J Nucl Med.

[CR11] Gafita A, Rauscher I, Fendler WP (2022). Measuring response in metastatic castration-resistant prostate cancer using PSMA PET/CT: comparison of RECIST 1.1, aPCWG3, aPERCIST, PPP, and RECIP 1.0 criteria. Eur J Nucl Med Mol Imaging.

[CR12] Hoberuck S, Lock S, Winzer R (2020). [(68)Ga]Ga-PSMA-11 PET before and after initial long-term androgen deprivation in patients with newly diagnosed prostate cancer: a retrospective single-center study. EJNMMI Res.

[CR13] Hofman MS, Lawrentschuk N, Francis RJ (2020). Prostate-specific membrane antigen PET-CT in patients with high-risk prostate cancer before curative-intent surgery or radiotherapy (proPSMA): a prospective, randomised, multicentre study. Lancet.

[CR14] Hope TA, Truillet C, Ehman EC (2017). 68Ga-PSMA-11 PET imaging of response to androgen receptor inhibition: first human experience. J Nucl Med.

[CR15] Lutje S, Slavik R, Fendler W, Herrmann K, Eiber M (2017). PSMA ligands in prostate cancer—probe optimization and theranostic applications. Methods.

[CR16] Onal C, Guler OC, Torun N, Reyhan M, Yapar AF (2020). The effect of androgen deprivation therapy on (68)Ga-PSMA tracer uptake in non-metastatic prostate cancer patients. Eur J Nucl Med Mol Imaging.

[CR17] Oruc Z, Guzel Y, Ebinc S (2021). Efficacy of 68Ga-PSMA PET/CT-derived whole-body volumetric parameters in predicting response to second-generation androgen receptor axis-targeted therapy, and the prognosis in metastatic hormone-refractory prostate cancer patients. Nucl Med Commun.

[CR18] Plouznikoff N, Artigas C, Sideris S (2019). Evaluation of PSMA expression changes on PET/CT before and after initiation of novel antiandrogen drugs (enzalutamide or abiraterone) in metastatic castration-resistant prostate cancer patients. Ann Nucl Med.

[CR19] Rosset A, Spadola L, Ratib O (2004). OsiriX: an open-source software for navigating in multidimensional DICOM images. J Digit Imaging.

[CR20] Schaeffer E, Srinivas S, Antonarakis ES (2021). NCCN guidelines insights: prostate cancer, version 1. 2021. J Natl Compr Canc Netw..

[CR21] Scher HI, Morris MJ, Stadler WM (2016). Trial design and objectives for castration-resistant prostate cancer: updated recommendations from the prostate cancer clinical trials working group 3. J Clin Oncol.

[CR22] Seifert R, Herrmann K, Kleesiek J (2020). Semiautomatically quantified tumor volume using (68)Ga-PSMA-11 PET as a biomarker for survival in patients with advanced prostate cancer. J Nucl Med.

[CR23] Silver DA, Pellicer I, Fair WR, Heston WD, Cordon-Cardo C (1997). Prostate-specific membrane antigen expression in normal and malignant human tissues. Clin Cancer Res.

[CR24] Sung H, Ferlay J, Siegel RL (2021). Global cancer statistics 2020: GLOBOCAN estimates of incidence and mortality worldwide for 36 cancers in 185 countries. CA Cancer J Clin.

[CR25] The Jamovi Project [computer program]. Version 1.6.23: Jamovi; 2021

[CR26] Tombal B, Borre M, Rathenborg P (2014). Enzalutamide monotherapy in hormone-naive prostate cancer: primary analysis of an open-label, single-arm, phase 2 study. Lancet Oncol.

[CR27] Tseng JR, Chang SH, Wu YY (2022). Impact of three-month androgen deprivation therapy on [68Ga]Ga-PSMA-11 PET/CT indices in men with advanced prostate cancer-results from a pilot prospective study. Cancers.

[CR28] Vaz S, Hadaschik B, Gabriel M, Herrmann K, Eiber M, Costa D (2020). Influence of androgen deprivation therapy on PSMA expression and PSMA-ligand PET imaging of prostate cancer patients. Eur J Nucl Med Mol Imaging.

[CR29] Wahl RL, Jacene H, Kasamon Y, Lodge MA (2009). From RECIST to PERCIST: evolving considerations for PET response criteria in solid tumors. J Nucl Med.

[CR30] Zukotynski KA, Emmenegger U, Hotte S (2021). prospective, single-arm trial evaluating changes in uptake patterns on prostate-specific membrane antigen-targeted (18)F-DCFPyL PET/CT in patients with castration-resistant prostate cancer starting abiraterone or enzalutamide. J Nucl Med.

