# Supplementary material for: Associations of quantitative whole-body PSMA-PET metrics with PSA progression status under long-term androgen deprivation therapy in prostate cancer patients: a retrospective single-center study
Source: Eur J Hybrid Imaging. 2023 Oct 2;7:18. doi: 10.1186/s41824-023-00178-1 (PMC10542625; doi:10.1186/s41824-023-00178-1)
Supplement: Supplementary file 1 — Additional file 1. Supplementary Materials. [file 41824_2023_178_MOESM1_ESM.docx]

| **Patient ID** | **Gleason Score (from prostatectomy when available)** | **Castration Status (1: CSPC; 2: CRPC)** | **RECIP 1.0 (PD vs. non-PD)** | **WB-PSMA-VOL % Change** | **WB-PSMA-SUV_mean_ % Change** |
| --- | --- | --- | --- | --- | --- |
| 1 | 3+3=6 | 2 | non-PD | -70% | -15% |
| 2 | 3+4=7 | 1 | PD | 1068% | 69% |
| 3 | 3+5=8 | 1 | non-PD | 0% | 0% |
| 4 | 3+4=7 | 1 | non-PD | -78% | -33% |
| 5 | 5+5=10 | 1 | PD | 2611% | 14% |
| 6 | 5+4=9 | 1 | non-PD | -92% | -39% |
| 7 | 4+5=9 | 1 | non-PD | -100% | -100% |
| 8 | 3+4=7 | 1 | non-PD | -100% | -100% |
| 9 | 4+4=8 | 2 | non-PD | 0% | 0% |
| 10 | 4+5=9 | 1 | non-PD | -96% | -8% |
| 11 | 5+4=9 | 1 | non-PD | -83% | -41% |
| 12 | 4+4=8 | 1 | non-PD | -17% | 60% |
| 13 | 4+4=8 | 1 | non-PD | -33% | -3% |
| 14 | 3+4=7 | 1 | non-PD | -33% | -12% |
| 15 | 3+4=7 | 1 | non-PD | 0% | 0% |
| 16 | 4+5=9 | 1 | non-PD | -92% | -21% |
| 17 | 5+4=9 | 1 | non-PD | -78% | -17% |
| 18 | 4+5=9 | 1 | non-PD | -100% | -100% |
| 19 | 4+4=8 | 1 | PD | 290% | -2% |
| 20 | 3+4=7 | 1 | non-PD | -93% | -15% |
| 21 | 3+4=7 | 1 | non-PD | -98% | -41% |
| 22 | 5+5=10 | 1 | non-PD | -100% | -100% |
| 23 | 4+4=8 | 2 | non-PD | -100% | -35% |
| 24 | 4+3=7 | 2 | non-PD | -100% | -100% |
| 25 | 5+5=10 | 2 | non-PD | 0% | 0% |
| 26 | 3+4=7 | 1 | non-PD | -94% | -7% |
| 27 | 5+5=10 | 1 | non-PD | -44% | 4% |
| 28 | 4+3=7 | 1 | PD | 206% | -3% |
| 29 | 3+3=6 | 1 | non-PD | -88% | -38% |
| 30 | 4+5=9 | 2 | non-PD | -67% | 72% |
| 31 | 4+5=9 | 1 | non-PD | -100% | -100% |
| 32 | 4+5=9 | 1 | PD | 4406% | 5260% |
| 33 | 4+5=9 | 1 | non-PD | -93% | -19% |
| 34 | 5+4=9 | 1 | non-PD | -100% | -100% |
| 35 | 5+4=9 | 1 | non-PD | -84% | -28% |

Patient-based reporting of Gleason Score, castration status, classification of PD vs. non-PD based on RECIP 1.0, and percent changes in WB-PSMA-VOL and WB-PSMA-SUV_mean_.
